# Di-μ-chlorido-dichlorido-bis{μ-6,6′-dimethoxy-2,2′-[*o*-phenylenebis(nitrilomethylidyne)]diphenolato}dilead(II)dizinc(II) *N*,*N*′-dimethyl­formamide disolvate

**DOI:** 10.1107/S1600536808032704

**Published:** 2008-10-15

**Authors:** Hailong Wang, Daopeng Zhang, Li-Fang Zhang

**Affiliations:** a School of Chemistry and Chemical Technology, Shandong University, Jinan 250100, People’s Republic of China

## Abstract

The title compound, [Pb_2_Zn_2_(C_22_H_18_N_2_O_4_)_2_Cl_4_]·2C_3_H_7_NO, was synthesized using a step-by-step method and has a slipped sandwich configuration. The coordination environment of the Zn^2+^ ion is distorted square-pyramidal and it is coordinated by N_2_O_2_ of the Schiff base ligand and chloride; each Pb^2+^ ion is coordinated by the four 6,6′-dimeth­oxy-2,2′-[*o*-phenyl­ene­bis(nitrilo­methyl­idyne)]diphenolate (*L*) O atoms and two chloride ions. The Zn^II^Pb^II^ dinuclear unit, through an inversion-symmetry operation, forms a tetra­meric complex with double chloride bridges.

## Related literature

For related literature, see: Karlin (1993 ▶); Korupoju *et al.* (2000 ▶); Lo *et al.* (2004 ▶); Ni *et al.* (2005 ▶); Sui *et al.* (2007 ▶); Ward (2007 ▶).
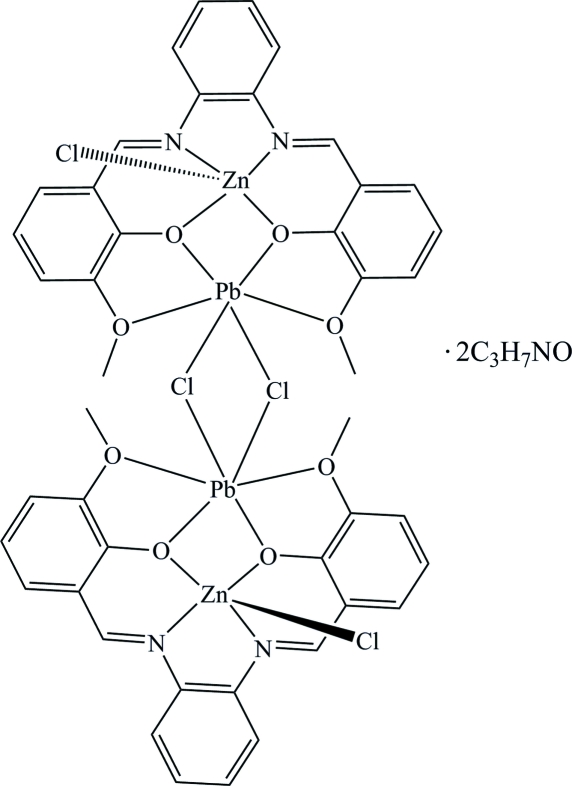


## Experimental

### 

#### Crystal data

[Pb_2_Zn_2_(C_22_H_18_N_2_O_4_)_2_Cl_4_]·2C_3_H_7_NO
*M* *_r_* = 1581.88
Monoclinic, 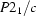

*a* = 7.4955 (6) Å
*b* = 32.119 (3) Å
*c* = 11.2366 (9) Å
β = 95.729 (2)°
*V* = 2691.7 (4) Å^3^
*Z* = 2
Mo *K*α radiation
μ = 7.38 mm^−1^
*T* = 295 (2) K
0.20 × 0.15 × 0.10 mm

#### Data collection

Bruker APEXII CCD area-detector diffractometer
Absorption correction: multi-scan (*SADABS*; Sheldrick, 2003 ▶) *T*_min_ = 0.320, *T*_max_ = 0.526 (expected range = 0.291–0.478)
13373 measured reflections
4719 independent reflections
3176 reflections with *I* > 2σ(*I*)
*R*_int_ = 0.063

#### Refinement

*R*[*F* ^2^ > 2σ(*F* ^2^)] = 0.038
*wR*(*F* ^2^) = 0.066
*S* = 0.93
4719 reflections
336 parameters
H-atom parameters constrained
Δρ_max_ = 0.55 e Å^−3^
Δρ_min_ = −0.64 e Å^−3^

### 

Data collection: *APEX2* (Bruker, 2004 ▶); cell refinement: *SAINT-Plus* (Bruker, 2001 ▶); data reduction: *SAINT-Plus*; program(s) used to solve structure: *SHELXS97* (Sheldrick, 2008 ▶); program(s) used to refine structure: *SHELXL97* (Sheldrick, 2008 ▶); molecular graphics: *XP* in *SHELXTL* (Sheldrick, 2008 ▶); software used to prepare material for publication: *XP* in *SHELXTL*.

## Supplementary Material

Crystal structure: contains datablocks global, I. DOI: 10.1107/S1600536808032704/kp2192sup1.cif

Structure factors: contains datablocks I. DOI: 10.1107/S1600536808032704/kp2192Isup2.hkl

Additional supplementary materials:  crystallographic information; 3D view; checkCIF report

## Figures and Tables

**Table:** 

N1—Zn1	2.062 (5)
N2—Zn1	2.064 (5)
O1—Pb1	2.720 (5)
O2—Zn1	2.026 (4)
O2—Pb1	2.408 (4)
O3—Zn1	2.025 (4)
O3—Pb1	2.386 (4)
O4—Pb1	2.690 (5)
Zn1—Cl1	2.2544 (18)
Pb1—Cl2	2.6138 (18)

**Table:** 

O3—Zn1—O2	80.49 (16)
O3—Zn1—N1	144.11 (18)
O2—Zn1—N1	88.19 (18)
O3—Zn1—N2	88.00 (18)
O2—Zn1—N2	141.06 (18)
N1—Zn1—N2	79.7 (2)
O3—Zn1—Cl1	107.95 (13)
O2—Zn1—Cl1	108.74 (13)
N1—Zn1—Cl1	107.94 (14)
N2—Zn1—Cl1	110.20 (15)
O3—Pb1—O2	66.18 (14)
O3—Pb1—Cl2	89.13 (11)
O2—Pb1—Cl2	91.86 (10)
O3—Pb1—O4	61.20 (14)
O2—Pb1—O4	127.37 (14)
Cl2—Pb1—O4	88.05 (11)
O3—Pb1—O1	126.94 (14)
O2—Pb1—O1	60.79 (14)
Cl2—Pb1—O1	94.14 (12)
O4—Pb1—O1	171.53 (14)

**Fig. 1.**
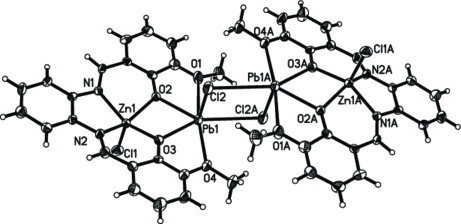
A view of (I) with the atom-labelling scheme. Displacement ellipsoids are drawn at the 30% probability level, solvate DMF molecules are ommited for clarity.

**Fig. 2.**
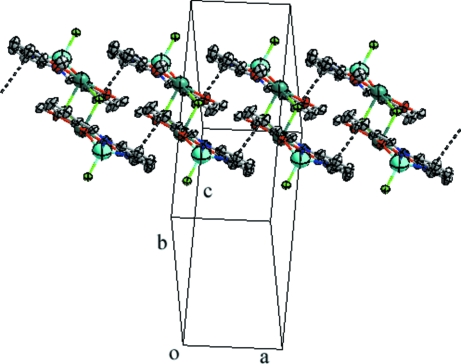
A view of crystal packing based on π–π interactions.

## supplementary crystallographic information

### Comment

Heterometallic complexes have been intensively studied owing to their unique physical and chemical properties (Ward *et al.*, 2007 and Ni *et al.*, 2005). In addition, these compounds exist at the active sites of many metalloenzymes and play important roles in biological systems (Karlin, 1993). It is necessary to extend the  application of heterometallic compounds. Herein, a novel heterometallic tetranuclear (Zn^II^Pb^II^)_2_ compound has been obtained using step-by-step method and its structure is depcited.

The compound I is a tetranuclear neutral complex with a slipped sandwich configuration (Fig. 1). Each Zn(II)  is coordinated in a square-pyramidal geometry with the basal square formed by two nitrogen atoms and two oxygen atoms from *L* ligand, with the apical position occupied by terminal chlorine atom. The coordination environment of each Pb(II)  is a distorted octahedral geometry composed of four oxygen atoms from ligand and two bridging chlorine atoms. Zn(II)  and Pb(II) are connected *via* two bridging oxygen atoms of the ligand, and two Pb(II) atoms are connected by two bridging chlorine atoms. The bond lengths of Zn—O, Zn—N and Zn—Cl are normal (Korupoju *et al.*, 2000). Through π-π interaction between the rings C9—C14 and C16—C21 [symmetry code; (i) -1 + *x*, *y*, *z*] with centroid distance of 3.730 (3) /A [*Cg*1··· *Cg*2^i^ ]  the discrete tetranuclear (Zn^II^Pb^II^)_2_ units forms a supramolecular structure (Fig. 2).

### Experimental

The H_2_L ligand and complex ZnL was synthesized according to the literature (Lo *et al.*, 2004; Sui *et al.* 2007). Synthesis of the compound I was obtained by allowing the mixure of ZnL (0.088 g, 0.2 mmol) and PbCl_2_.2H_2_O(0.063 g, 0.2 mmol)  to be refluxed in the DMF solution, cooled down to room temperature, then filtered, and suitable yellow crystals were obtained by slow evaporation of the filtrate at room temperature (yield: about 45%).

### Refinement

All H-atoms bound to carbon were refined using a riding model with distance C—H = 0.93 Å, *U*_iso_ = 1.2*U*_eq_ (C) for aromatic atoms and C—H = 0.96 Å, *U*_iso_ = 1.5*U*_eq_ (C) for methyl atoms.

### Crystal data

**Table:**

[Zn_2_Pb_2_(C_22_H_18_N_2_O_4_)_2_Cl_4_]·2C_3_H_7_NO	*F*(000) = 1528
*M**_r_* = 1581.88	*D*_x_ = 1.952 Mg m^−^^3^
Monoclinic, *P*2_1_/*c*	Mo *K*α radiation, λ = 0.71073 Å
Hall symbol: -P 2ybc	Cell parameters from 3748 reflections
*a* = 7.4955 (6) Å	θ = 1.9–26.5°
*b* = 32.119 (3) Å	µ = 7.38 mm^−^^1^
*c* = 11.2366 (9) Å	*T* = 295 K
β = 95.729 (2)°	Block, yellow
*V* = 2691.7 (4) Å^3^	0.20 × 0.15 × 0.10 mm
*Z* = 2	

### Data collection

**Table:**

Bruker APEXII CCD area-detector diffractometer	4719 independent reflections
Radiation source: fine-focus sealed tube	3176 reflections with *I* > 2σ(*I*)
graphite	*R*_int_ = 0.063
Detector resolution: 0 pixels mm^-1^	θ_max_ = 25.0°, θ_min_ = 1.9°
φ and ω scans	*h* = −8→7
Absorption correction: multi-scan (SADABS; Sheldrick, 2003)	*k* = −38→37
*T*_min_ = 0.320, *T*_max_ = 0.526	*l* = −13→12
13373 measured reflections	

### Refinement

**Table:**

Refinement on *F*^2^	Primary atom site location: structure-invariant direct methods
Least-squares matrix: full	Secondary atom site location: difference Fourier map
*R*[*F*^2^ > 2σ(*F*^2^)] = 0.038	Hydrogen site location: inferred from neighbouring sites
*wR*(*F*^2^) = 0.066	H-atom parameters constrained
*S* = 0.93	*w* = 1/[σ^2^(*F*_o_^2^) + (0.017*P*)^2^] where *P* = (*F*_o_^2^ + 2*F*_c_^2^)/3
4719 reflections	(Δ/σ)_max_ = 0.001
336 parameters	Δρ_max_ = 0.55 e Å^−^^3^
0 restraints	Δρ_min_ = −0.64 e Å^−^^3^

### Special details

**Table:**

Geometry. All e.s.d.'s (except the e.s.d. in the dihedral angle between two l.s. planes) are estimated using the full covariance matrix. The cell e.s.d.'s are taken into account individually in the estimation of e.s.d.'s in distances, angles and torsion angles; correlations between e.s.d.'s in cell parameters are only used when they are defined by crystal symmetry. An approximate (isotropic) treatment of cell e.s.d.'s is used for estimating e.s.d.'s involving l.s. planes.
Refinement. Refinement of *F*^2^ against ALL reflections. The weighted *R*-factor *wR* and goodness of fit *S* are based on *F*^2^, conventional *R*-factors *R* are based on *F*, with *F* set to zero for negative *F*^2^. The threshold expression of *F*^2^ > σ(*F*^2^) is used only for calculating *R*-factors(gt) *etc*. and is not relevant to the choice of reflections for refinement. *R*-factors based on *F*^2^ are statistically about twice as large as those based on *F*, and *R*- factors based on ALL data will be even larger.

### Fractional atomic coordinates and isotropic or equivalent isotropic displacement parameters (Å^2^)

**Table:**

	*x*	*y*	*z*	*U*_iso_*/*U*_eq_	
C1	1.1566 (10)	−0.0054 (2)	0.1667 (6)	0.067 (2)	
H1A	1.0503	−0.0177	0.1926	0.100*	
H1B	1.2482	−0.0263	0.1655	0.100*	
H1C	1.1300	0.0059	0.0879	0.100*	
C2	1.3705 (10)	0.0487 (2)	0.2264 (6)	0.0448 (19)	
C3	1.4939 (10)	0.0359 (2)	0.1533 (6)	0.054 (2)	
H3	1.4754	0.0109	0.1118	0.065*	
C4	1.6467 (10)	0.0591 (2)	0.1391 (6)	0.057 (2)	
H4	1.7287	0.0500	0.0878	0.068*	
C5	1.6746 (9)	0.0953 (2)	0.2017 (6)	0.0458 (19)	
H5	1.7778	0.1106	0.1932	0.055*	
C6	1.5510 (8)	0.11026 (19)	0.2791 (5)	0.0309 (16)	
C7	1.3931 (9)	0.0871 (2)	0.2908 (5)	0.0339 (16)	
C8	1.5982 (8)	0.14809 (19)	0.3455 (6)	0.0349 (17)	
H8	1.7141	0.1582	0.3433	0.042*	
C9	1.5514 (8)	0.20447 (19)	0.4732 (5)	0.0308 (16)	
C10	1.7104 (8)	0.22575 (19)	0.4619 (6)	0.0350 (17)	
H10	1.7875	0.2167	0.4073	0.042*	
C11	1.7534 (10)	0.2602 (2)	0.5317 (6)	0.048 (2)	
H11	1.8607	0.2740	0.5244	0.058*	
C12	1.6408 (10)	0.2745 (2)	0.6123 (6)	0.052 (2)	
H12	1.6722	0.2978	0.6589	0.062*	
C13	1.4798 (9)	0.2540 (2)	0.6236 (6)	0.0427 (19)	
H13	1.4019	0.2641	0.6763	0.051*	
C14	1.4350 (8)	0.21857 (19)	0.5565 (5)	0.0303 (16)	
C15	1.1756 (9)	0.1993 (2)	0.6481 (6)	0.0408 (18)	
H15	1.2067	0.2202	0.7036	0.049*	
C16	1.0212 (9)	0.1750 (2)	0.6657 (6)	0.0363 (17)	
C17	0.9185 (10)	0.1873 (2)	0.7569 (6)	0.057 (2)	
H17	0.9537	0.2107	0.8020	0.069*	
C18	0.7699 (11)	0.1665 (3)	0.7822 (7)	0.073 (3)	
H18	0.7050	0.1757	0.8434	0.088*	
C19	0.7150 (10)	0.1318 (2)	0.7177 (7)	0.059 (2)	
H19	0.6137	0.1173	0.7360	0.071*	
C20	0.8083 (9)	0.1183 (2)	0.6266 (6)	0.0422 (18)	
C21	0.9628 (8)	0.13985 (19)	0.5965 (6)	0.0322 (16)	
C22	0.5984 (9)	0.0631 (2)	0.5684 (6)	0.059 (2)	
H22A	0.5849	0.0400	0.5142	0.088*	
H22B	0.5006	0.0821	0.5510	0.088*	
H22C	0.5987	0.0532	0.6491	0.088*	
C23	1.2723 (12)	0.0874 (3)	0.8580 (7)	0.087 (3)	
H23A	1.3962	0.0798	0.8745	0.130*	
H23B	1.2543	0.1010	0.7817	0.130*	
H23C	1.1994	0.0628	0.8568	0.130*	
C24	1.3274 (12)	0.1533 (3)	0.9737 (8)	0.092 (3)	
H24A	1.4516	0.1459	0.9891	0.138*	
H24B	1.2899	0.1678	1.0417	0.138*	
H24C	1.3113	0.1710	0.9046	0.138*	
C25	1.0834 (13)	0.1067 (3)	1.0144 (8)	0.082 (3)	
H25	1.0538	0.1253	1.0729	0.099*	
N1	1.4929 (7)	0.16881 (15)	0.4072 (4)	0.0302 (12)	
N2	1.2753 (7)	0.19497 (15)	0.5626 (4)	0.0341 (13)	
N3	1.2211 (9)	0.1160 (2)	0.9524 (6)	0.069 (2)	
O1	1.2183 (7)	0.02728 (14)	0.2479 (4)	0.0568 (14)	
O2	1.2672 (5)	0.09841 (12)	0.3583 (4)	0.0358 (11)	
O3	1.0450 (6)	0.12607 (12)	0.5055 (4)	0.0411 (12)	
O4	0.7656 (6)	0.08411 (14)	0.5550 (4)	0.0484 (13)	
O5	0.9982 (10)	0.0763 (2)	0.9986 (7)	0.126 (3)	
Zn1	1.22386 (10)	0.15710 (2)	0.41482 (7)	0.0330 (2)	
Pb1	1.00874 (4)	0.060131 (8)	0.40830 (2)	0.04083 (10)	
Cl1	1.0695 (2)	0.19248 (6)	0.26480 (16)	0.0515 (5)	
Cl2	1.1909 (2)	0.02574 (6)	0.59314 (17)	0.0555 (5)	

### Atomic displacement parameters (Å^2^)

**Table:**

	*U*^11^	*U*^22^	*U*^33^	*U*^12^	*U*^13^	*U*^23^
C1	0.079 (6)	0.048 (5)	0.072 (6)	−0.013 (4)	−0.002 (5)	−0.025 (5)
C2	0.053 (5)	0.046 (5)	0.037 (4)	0.000 (4)	0.012 (4)	−0.010 (4)
C3	0.062 (6)	0.042 (5)	0.059 (5)	−0.006 (4)	0.016 (5)	−0.025 (4)
C4	0.057 (5)	0.058 (5)	0.058 (5)	0.003 (4)	0.024 (4)	−0.027 (5)
C5	0.034 (4)	0.061 (5)	0.043 (5)	0.006 (4)	0.008 (4)	−0.001 (4)
C6	0.034 (4)	0.029 (4)	0.032 (4)	0.005 (3)	0.011 (3)	0.002 (3)
C7	0.038 (4)	0.033 (4)	0.029 (4)	0.008 (3)	0.000 (3)	0.005 (3)
C8	0.027 (4)	0.032 (4)	0.046 (4)	−0.006 (3)	0.006 (4)	0.007 (3)
C9	0.025 (4)	0.037 (4)	0.031 (4)	0.003 (3)	0.000 (3)	0.002 (3)
C10	0.029 (4)	0.039 (4)	0.036 (4)	−0.004 (3)	0.002 (3)	−0.002 (3)
C11	0.045 (5)	0.043 (5)	0.054 (5)	−0.016 (4)	−0.004 (4)	−0.002 (4)
C12	0.051 (5)	0.051 (5)	0.054 (5)	−0.007 (4)	0.012 (4)	−0.014 (4)
C13	0.042 (5)	0.038 (4)	0.049 (5)	0.002 (3)	0.009 (4)	0.000 (4)
C14	0.030 (4)	0.030 (4)	0.030 (4)	−0.002 (3)	−0.004 (3)	0.003 (3)
C15	0.046 (5)	0.038 (4)	0.040 (4)	−0.006 (3)	0.012 (4)	0.003 (3)
C16	0.043 (4)	0.035 (4)	0.033 (4)	−0.001 (3)	0.016 (4)	0.002 (3)
C17	0.057 (5)	0.053 (5)	0.065 (6)	−0.007 (4)	0.025 (5)	−0.018 (4)
C18	0.076 (6)	0.073 (7)	0.081 (7)	−0.017 (5)	0.056 (5)	−0.017 (5)
C19	0.047 (5)	0.063 (6)	0.073 (6)	−0.014 (4)	0.031 (5)	−0.007 (5)
C20	0.035 (4)	0.046 (5)	0.046 (5)	−0.004 (3)	0.009 (4)	0.003 (4)
C21	0.026 (4)	0.034 (4)	0.038 (4)	0.005 (3)	0.011 (3)	−0.004 (3)
C22	0.050 (5)	0.063 (5)	0.063 (5)	−0.020 (4)	0.010 (4)	0.012 (4)
C23	0.084 (7)	0.093 (7)	0.088 (7)	−0.016 (6)	0.037 (6)	−0.029 (6)
C24	0.101 (8)	0.070 (7)	0.101 (8)	−0.049 (6)	−0.007 (6)	−0.013 (6)
C25	0.089 (8)	0.074 (7)	0.090 (7)	−0.029 (6)	0.043 (6)	−0.018 (6)
N1	0.029 (3)	0.029 (3)	0.033 (3)	0.000 (3)	0.007 (3)	−0.001 (3)
N2	0.032 (3)	0.034 (3)	0.038 (3)	−0.006 (2)	0.011 (3)	−0.001 (3)
N3	0.067 (5)	0.073 (5)	0.068 (5)	−0.018 (4)	0.017 (4)	−0.010 (4)
O1	0.069 (4)	0.044 (3)	0.060 (3)	−0.015 (3)	0.018 (3)	−0.023 (3)
O2	0.034 (3)	0.030 (3)	0.045 (3)	−0.002 (2)	0.013 (2)	−0.005 (2)
O3	0.043 (3)	0.033 (3)	0.051 (3)	−0.003 (2)	0.023 (2)	−0.003 (2)
O4	0.035 (3)	0.046 (3)	0.065 (3)	−0.012 (2)	0.013 (3)	−0.004 (3)
O5	0.115 (6)	0.114 (7)	0.158 (7)	−0.039 (5)	0.063 (5)	−0.014 (5)
Zn1	0.0303 (4)	0.0303 (4)	0.0393 (5)	−0.0020 (3)	0.0080 (4)	−0.0011 (4)
Pb1	0.03765 (16)	0.03548 (16)	0.05010 (18)	−0.00722 (14)	0.00808 (13)	−0.00237 (15)
Cl1	0.0443 (11)	0.0581 (13)	0.0522 (12)	0.0087 (9)	0.0043 (10)	0.0121 (10)
Cl2	0.0504 (12)	0.0521 (12)	0.0621 (13)	−0.0162 (9)	−0.0032 (10)	0.0058 (10)

### Geometric parameters (Å, °)

**Table:**

C1—O1	1.437 (7)	C17—C18	1.354 (10)
C1—H1A	0.9600	C17—H17	0.9300
C1—H1B	0.9600	C18—C19	1.370 (9)
C1—H1C	0.9600	C18—H18	0.9300
C2—C3	1.361 (9)	C19—C20	1.367 (9)
C2—O1	1.373 (8)	C19—H19	0.9300
C2—C7	1.432 (8)	C20—O4	1.379 (7)
C3—C4	1.389 (9)	C20—C21	1.420 (8)
C3—H3	0.9300	C21—O3	1.321 (7)
C4—C5	1.365 (9)	C22—O4	1.444 (7)
C4—H4	0.9300	C22—H22A	0.9600
C5—C6	1.416 (8)	C22—H22B	0.9600
C5—H5	0.9300	C22—H22C	0.9600
C6—C7	1.415 (8)	C23—N3	1.483 (9)
C6—C8	1.451 (8)	C23—H23A	0.9600
C7—O2	1.319 (7)	C23—H23B	0.9600
C8—N1	1.286 (7)	C23—H23C	0.9600
C8—H8	0.9300	C24—N3	1.446 (9)
C9—C10	1.391 (8)	C24—H24A	0.9600
C9—N1	1.411 (7)	C24—H24B	0.9600
C9—C14	1.416 (8)	C24—H24C	0.9600
C10—C11	1.375 (8)	C25—O5	1.172 (9)
C10—H10	0.9300	C25—N3	1.335 (10)
C11—C12	1.377 (9)	C25—H25	0.9300
C11—H11	0.9300	N1—Zn1	2.062 (5)
C12—C13	1.392 (9)	N2—Zn1	2.064 (5)
C12—H12	0.9300	O1—Pb1	2.720 (5)
C13—C14	1.389 (8)	O2—Zn1	2.026 (4)
C13—H13	0.9300	O2—Pb1	2.408 (4)
C14—N2	1.424 (7)	O3—Zn1	2.025 (4)
C15—N2	1.283 (7)	O3—Pb1	2.386 (4)
C15—C16	1.426 (8)	O4—Pb1	2.690 (5)
C15—H15	0.9300	Zn1—Cl1	2.2544 (18)
C16—C17	1.399 (9)	Pb1—Cl2	2.6138 (18)
C16—C21	1.416 (8)		
			
O1—C1—H1A	109.5	O3—C21—C20	118.4 (6)
O1—C1—H1B	109.5	C16—C21—C20	118.2 (6)
H1A—C1—H1B	109.5	O4—C22—H22A	109.5
O1—C1—H1C	109.5	O4—C22—H22B	109.5
H1A—C1—H1C	109.5	H22A—C22—H22B	109.5
H1B—C1—H1C	109.5	O4—C22—H22C	109.5
C3—C2—O1	125.5 (7)	H22A—C22—H22C	109.5
C3—C2—C7	120.8 (7)	H22B—C22—H22C	109.5
O1—C2—C7	113.7 (6)	N3—C23—H23A	109.5
C2—C3—C4	121.7 (7)	N3—C23—H23B	109.5
C2—C3—H3	119.2	H23A—C23—H23B	109.5
C4—C3—H3	119.2	N3—C23—H23C	109.5
C5—C4—C3	118.9 (7)	H23A—C23—H23C	109.5
C5—C4—H4	120.5	H23B—C23—H23C	109.5
C3—C4—H4	120.5	N3—C24—H24A	109.5
C4—C5—C6	122.0 (7)	N3—C24—H24B	109.5
C4—C5—H5	119.0	H24A—C24—H24B	109.5
C6—C5—H5	119.0	N3—C24—H24C	109.5
C7—C6—C5	118.9 (6)	H24A—C24—H24C	109.5
C7—C6—C8	123.7 (6)	H24B—C24—H24C	109.5
C5—C6—C8	117.3 (6)	O5—C25—N3	123.1 (10)
O2—C7—C6	124.2 (6)	O5—C25—H25	118.5
O2—C7—C2	118.2 (6)	N3—C25—H25	118.5
C6—C7—C2	117.6 (6)	C8—N1—C9	121.9 (5)
N1—C8—C6	125.4 (6)	C8—N1—Zn1	125.7 (4)
N1—C8—H8	117.3	C9—N1—Zn1	112.3 (4)
C6—C8—H8	117.3	C15—N2—C14	122.3 (6)
C10—C9—N1	125.1 (6)	C15—N2—Zn1	126.2 (5)
C10—C9—C14	119.6 (6)	C14—N2—Zn1	111.4 (4)
N1—C9—C14	115.3 (5)	C25—N3—C24	122.8 (8)
C11—C10—C9	119.8 (6)	C25—N3—C23	120.2 (7)
C11—C10—H10	120.1	C24—N3—C23	117.1 (7)
C9—C10—H10	120.1	C2—O1—C1	118.4 (5)
C10—C11—C12	121.3 (7)	C2—O1—Pb1	117.8 (4)
C10—C11—H11	119.3	C1—O1—Pb1	122.2 (4)
C12—C11—H11	119.3	C7—O2—Zn1	125.6 (4)
C11—C12—C13	119.7 (7)	C7—O2—Pb1	129.2 (4)
C11—C12—H12	120.1	Zn1—O2—Pb1	104.13 (17)
C13—C12—H12	120.1	C21—O3—Zn1	127.4 (4)
C14—C13—C12	120.2 (7)	C21—O3—Pb1	127.7 (4)
C14—C13—H13	119.9	Zn1—O3—Pb1	104.90 (17)
C12—C13—H13	119.9	C20—O4—C22	117.7 (5)
C13—C14—C9	119.3 (6)	C20—O4—Pb1	117.0 (4)
C13—C14—N2	124.7 (6)	C22—O4—Pb1	125.3 (4)
C9—C14—N2	116.0 (5)	O3—Zn1—O2	80.49 (16)
N2—C15—C16	125.8 (6)	O3—Zn1—N1	144.11 (18)
N2—C15—H15	117.1	O2—Zn1—N1	88.19 (18)
C16—C15—H15	117.1	O3—Zn1—N2	88.00 (18)
C17—C16—C21	117.8 (6)	O2—Zn1—N2	141.06 (18)
C17—C16—C15	117.4 (6)	N1—Zn1—N2	79.7 (2)
C21—C16—C15	124.8 (6)	O3—Zn1—Cl1	107.95 (13)
C18—C17—C16	122.6 (7)	O2—Zn1—Cl1	108.74 (13)
C18—C17—H17	118.7	N1—Zn1—Cl1	107.94 (14)
C16—C17—H17	118.7	N2—Zn1—Cl1	110.20 (15)
C17—C18—C19	120.1 (7)	O3—Pb1—O2	66.18 (14)
C17—C18—H18	120.0	O3—Pb1—Cl2	89.13 (11)
C19—C18—H18	120.0	O2—Pb1—Cl2	91.86 (10)
C20—C19—C18	120.3 (7)	O3—Pb1—O4	61.20 (14)
C20—C19—H19	119.8	O2—Pb1—O4	127.37 (14)
C18—C19—H19	119.8	Cl2—Pb1—O4	88.05 (11)
C19—C20—O4	125.7 (7)	O3—Pb1—O1	126.94 (14)
C19—C20—C21	121.0 (7)	O2—Pb1—O1	60.79 (14)
O4—C20—C21	113.4 (6)	Cl2—Pb1—O1	94.14 (12)
O3—C21—C16	123.4 (6)	O4—Pb1—O1	171.53 (14)
